# A case series report of cancer patients undergoing group body psychotherapy

**DOI:** 10.12688/f1000research.12262.2

**Published:** 2018-09-05

**Authors:** Astrid Grossert, Gunther Meinlschmidt, Rainer Schaefert

**Affiliations:** 1 Department of Medical Oncology, University Hospital Basel, Basel, Switzerland; 2 Department of Psychosomatic Medicine, University Hospital Basel, Basel, Switzerland; 3 Faculty of Medicine, Ruhr-University Bochum, Bochum, Germany; 4 Faculty of Medicine, University of Basel, Basel, Switzerland; 5 Division of Clinical Psychology and Epidemiology, Department of Psychology, University of Basel, Basel, Switzerland; 6 Division of Clinical Psychology and Cognitive Behavioral Therapy, International Psychoanalytic University, Berlin, Germany

**Keywords:** body image, body integrity, body therapy, case report, group psychotherapy, malignant neoplasm, movement therapy, tumor

## Abstract

***Background**:* Disturbances in bodily wellbeing represent a key source of psychosocial suffering and impairment related to cancer. Therefore, interventions to improve bodily wellbeing in post-treatment cancer patients are of paramount importance. Notably, body psychotherapy (BPT) has been shown to improve bodily wellbeing in subjects suffering from a variety of mental disorders. However, how post-treatment cancer patients perceive and subjectively react to group BPT aiming at improving bodily disturbances has, to the best of our knowledge, not yet been described.

***Methods**:* We report on six patients undergoing outpatient group BPT that followed oncological treatment for malignant neoplasms. The BPT consisted of six sessions based on a scientific embodiment approach, integrating body-oriented techniques to improve patients’ awareness, perception, acceptance, and expression regarding their body.

***Results**:* The BPT was well accepted by all patients. Despite having undergone different types of oncological treatment for different cancer types and locations, all subjects reported having appreciated BPT and improved how they perceived their bodies. However, individual descriptions of improvements showed substantial heterogeneity across subjects. Notably, most patients indicated that sensations, perceptions, and other mental activities related to their own body intensified when proceeding through the group BPT sessions.

***Conclusion**:* The findings from this case series encourage and inform future studies examining whether group BPT is efficacious in post-treatment cancer patients and investigating the related mechanisms of action. The observed heterogeneity in individual descriptions of perceived treatment effects point to the need for selecting comprehensive indicators of changes in disturbances of bodily wellbeing as the primary patient-reported outcome in future clinical trials. While increases in mental activities related to their own body are commonly interpreted as important mechanisms of therapeutic action in BPT, follow-up assessments are needed to evaluate intended and unintended consequences of these changes in cancer patients.

## Introduction

Cancer is related to high individual and societal burden worldwide, which is caused not only by mortality, but also morbidity and impairment, as indicated by recent analyses in the context of the Global Burden of Disease study and other consortia ^1– 3^. Notably, while a significant proportion of cancer-related burden directly originates from the neoplasm and its treatment, psychosocial impairment represents another substantial aspect of cancer-related burden, which is triggered by the experiences and suffering related to cancer, that often persists beyond successful treatment of the tumor itself ^4– 8^.

Disturbances in bodily wellbeing represent one key aspect of these psychosocial impairments related to cancer ^9, 10^. Notably, the concept of cancer-related disturbances in bodily wellbeing and related constructs, such as perceived body integrity and body image, have been present for a long time, but with varying and sometimes conflicting definitions ^10– 13^. In a recent report and analysis, Rhoten examined the concept of ‘body image disturbances’ in the context of cancer. She identified three relevant attributes of body image disturbance: (1) self-perception of a change in appearance and displeasure with the change or perceived change in appearance; (2) decline in an area of function; and (3) psychological distress regarding changes in appearance and/or function ^12^. In line with these attributes, others have stressed that body image is a multidimensional construct, including elements such as perceptions, feelings, and attitudes toward the body, with body image disturbances being highly prevalent in cancer patients ^10^. The broader term ‘disturbances in bodily wellbeing’ (or ‘bodily disturbances’) acknowledges the fact that subjective bodily disturbances related to cancer are multifaceted, including key aspects often subsumed under the term ‘body image disturbance’, such as disturbances and related distress in perceptions, feelings, and attitudes toward the body, as well as in subjective appearance and/or function of the body. The concept of bodily disturbances builds on the understanding of the polar relations between bodily and mental processes and experiences. This body-mind perspective has gained considerable attention over the past decade within the embodied cognition or embodiment approach ^14^. In the context of the interventions described in this article, we put experiencing of the body in focus as a central pathway to experiencing and regulating the self, and regard the human being as a subjectively experiencing and embodied acting being that creates meaning in relation to the world ^15, 16^.

Health-related quality of life improvements in cancer patients are typically intended to be achieved using medical interventions, such as the provision of cytotoxic agents ^8^. Additionally, psychosocial interventions, including psychotherapy, for cancer patients and their caregivers have gained considerable attention in the past years ^17^. However, meta-analyses of their efficacy have yielded mixed results, with some suggestive of rather small or no effects associated with classical interventions, such as cognitive behavioral therapy, which are well-established and largely effective in many non-cancer contexts ^18– 21^. With regard to interventions that act via movement, there is ample evidence that physical activity and exercise i) are safe and feasible for cancer patients, ii) are linked to reduced cancer incidence (prevention), and iii) improve the quality of life and function of cancer patients, but with mostly small effects ^22– 26^.

Furthermore, the past several years have seen a renaissance of interventions focusing on existential topics, such as meaning, which have been applied to patients with a variety of life-threatening diseases (e.g., cancer ^27– 32^), including ‘Meaning-Centered Group Psychotherapy’, with the aim of helping patients with advanced cancer sustain or enhance a sense of meaning, peace and purpose in their lives ^33^. There is some evidence that body psychotherapy (BPT), defined as ‘psychotherapeutic treatment of mental disease or suffering, concomitantly using bodily and mental psychotherapeutic means’ ^34^, is efficacious for the treatment of different mental disorders ^35– 38^.

However, to the best of our knowledge, there are no data on the application of BPT in the context of cancer. Notably, given that BPT explicitly targets bodily aspects, such as perceptions, feelings, and attitudes toward the body, that are of paramount importance in the context of bodily disturbances in cancer patients (see above), elucidating BPT as an intervention to reduce disturbances of bodily wellbeing appears to be highly promising.

To conclude, disturbances of bodily wellbeing are highly prevalent in subjects with malignant neoplasm, and wellbeing often subsides despite successful interventions targeting the tumor itself. Therefore, identifying how to treat disturbances of bodily wellbeing in post-treatment cancer patients and therapeutic mechanisms is highly warranted, with BPT representing a highly promising and innovative approach.

To provide insight into the potential use of BPT in post-treatment cancer patients and to inform potential future clinical trials on this topic, we report on a series of six post-treatment cancer patients receiving group BPT aiming at improving bodily disturbances, focusing on how the patients perceived and subjectively reacted to the intervention. These patients are unique, and, to the best of our knowledge, these are the first published reports on the application of BPT in the context of cancer.

## Methods

### Therapeutic intervention

BPT was conducted by the first author of this case series (female, age = 35 years, Swiss citizen, trained psychologist and physiotherapist, four years of postgraduate training in body psychotherapy) and took place between October and December 2016. We describe here the key features of the intervention (structure of the description based on the ‘reporting recommendations for group-based behavior-change interventions’ ^39^ enriched by relevant elements from the ‘Template for Intervention Description and Replication (TIDieR) checklist and guide’ ^40^).

BPT represents an experience-oriented approach ^34, 41, 42^, grounded in the notion that bodily and mental experiences and processes (including more existential topics, such as meaning in life) are closely and mutually related. More specifically, BPT takes advantage of the embodied, enactive and environmentally embedded nature of basic cognition, emotion, intersubjectivity, and experiencing ^43^. In line with recently developed group body psychotherapy manuals for mental disorders (e.g., somatoform disorder, depression) that have been derived from disorder-specific intervention strategies ^37, 44– 46^, our group BPT followed a general BPT framework. Therefore, it focused on specific pathological processes of key relevance in post-treatment cancer patients when applying body-oriented techniques to improve patients’ bodily awareness, perception, emotional connectedness, acceptance, and expression ^41^.

The overall *goal* of this group BPT was to resolve bodily disturbances that are caused or triggered by the antecedent cancer and related treatments. Therefore, the group BPT aimed at supporting patients in learning to cope with untoward bodily sensations, feelings, and disturbances, such as changes in overt body image ^47, 48^, as well as changes in attitudes toward and perceptions of their own body ^49^, including feelings of insecurity and vulnerability ^50– 52^, stigmatization ^53^, impaired functioning ^52, 54^, and feelings of disconnectedness from one’s own body ^51^.

With regard to the *setting*, the BPT group (six participants) was provided under the auspices of the *Krebsliga Beider Basel* at their facilities. The group BPT consisted of six sessions of approximately 90 minutes each.

We chose a *group setting* because it offers advantages over individual therapy, including facilitation of specific therapeutic factors, such as vicarious learning and economic benefits ^55, 56^, in the absence of strong evidence suggesting clear superiority in outcomes of one setting over the other when comparing group and individual psychotherapies ^57– 59^.

The six sessions covered the following *topics*:

1) General introduction, fostering of group cohesion and focus on bodily perception;
2) Focus on bodily resources and grounding;
3) Focus on closeness and distance regulation;
4) Focus on social interactions and bodily impulses;
5) Focus on embodied emotions; and
6) Summary and transfer session.

*Sequencing* of the sessions was fixed, and every session consisted of four parts:

A) Introduction: Brief bodily exercise and exchange, preparing the specific topic of the session; review of the home task assigned during the past session, where appropriate;
B) Exercise: Psycho-educational element and an exercise triggering embodied experiences, focusing on the specific topic of the session;
C) Sharing: Exchange of experiences;
D) Closing: Résumé and farewell, hometask assignment, and outlook.

An outline of the content of each group BPT session is provided as Supplemental material (see Supplementary File 1). The execution of each session was tailored to the composition of the patient group and respective needs, acknowledging group processes that need to be addressed in connection with the content of each session.

*The tools* used during the sessions included materials, such as mats, ropes and balls.

The group BPT sessions were preceded by initial individual sessions (one per patient, maximum duration of 50 minutes) that were structured and documented using the basis documentation for Psycho-Oncology (PO-Bado) ^60^, assessing sociodemographics, medical history, main symptoms, previous experiences, and individual core topics of relevance within the scope of the intervention. Additionally, validated questionnaires assessing distress and bodily wellbeing (German version of the Body Appreciation Scale [BAS]) were applied ^61, 62^.

The patients were offered an additional facultative individual consultation session (one per patient, maximum duration of 50 minutes) following the last group BPT session to address open questions or to ensure ongoing therapeutic support.

### Data collection and integration

The group leader took written notes of key statements of the participants during and immediately after the six group therapy sessions. To collect information on the patients’ perspective of the therapeutic outcome of this group BPT, the participants provided written feedback at the end of the six sessions, focusing on the following topics: i) perceived changes, including subjective changes related to the perception of their own bodies; ii) group climate and cohesion; and iii) possibilities of creating new personal ties.

The data and collected information were integrated as follows: The key statements of the participants, enriched by information collected via PO-Bado, were sorted by the first author according to identified themes. These themes were derived from i) distress categories provided by the PO-Bado, and from ii) the list of psychosocial problems provided by the distress thermometer. This was complemented by iii) a resource perspective, and iv) common themes related to bodily experiences, focusing on the main topics of the respective sessions. The statements were then summarized, evaluated, and interpreted from a clinician perspective.

## Results

### Patient characteristics

**Figure 1.**
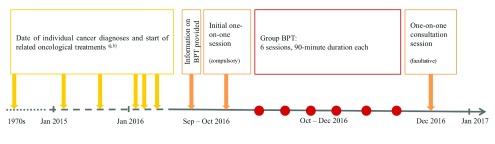
Timeline of cancer diagnoses in group BPT participants. BPT, body psychotherapy; Dec, December; Jan, January; Oct, October; Sep, September. ^a)^ Two patients were still receiving hormonal treatment while they were attending the group body psychotherapy sessions.

Information on the six patients, including demographic and other patient-specific information, main symptoms and concerns, medical and psychosocial history, past oncological interventions and current cancer disease status, is provided in Table 1. The timeline of initial cancer diagnosis, medical treatment and BPT is depicted in Figure 1. Notably, there were two other post-treatment cancer patients who were interested in participating in the BPT, but ultimately did not participate in the intervention (no further information is provided here because no informed consent to report on their cases in scientific publications was obtained from these subjects). Of these eight patients (six participating and two non-participating in the group BPT), five were informed of the group BPT service by the *Krebsliga Beider Basel,* and three patients were informed of the group by the Department of Psychosomatics at the University Hospital Basel.

**Table 1.** Information on socio-demographics, symptoms, medical history, and psychosocial history of the individual patients.

Patient	A	B	C	D	E	F
**Socio-demographics**						
Age (range [years])	51–75	25–50	25–50	51–75	51–75	25–50
Gender (female, male)	female	male	female	male	female	female
Current relationship (yes, no)	no	no	no	yes	yes	yes
Children (yes, no)	yes	yes	no	yes	no	yes
**Main symptoms of distress**						
Overall distress ^a)^	5	3	8	2	6	8
Fatigue/tiredness ^b)^	2	1	2	2	4	3
Mood swings ^b)^	3	2	3	4	3	3
Anxiety/worry/tension ^b)^	0	1	3	3	4	4
Depression/grief ^b)^	4	0	4	3	4	2
Impairment in daily activities ^b)^	1	1	2	2	3	3
Other psychological problems ^b)^	2	1	3	2	3	4
ECOG ^c)^	0	0	0	0	1	0
**Psychosocial treaments**						
Current professional psychosocial support	yes	yes	yes	yes	no	yes
Current psychoactive medication/opiates	yes	unknown	no	unknown	no	no
Previous psychological/ psychiatric treatment	no	unknown	no	yes	yes	yes
**Medical history**						
Type/localization of tumor	breast cancer, left	diffuse large B cell lymphoma, mediastinal	bladder cancer	seminoma, right, and prostata tumor	breast cancer, left	breast cancer, right
Metastases	no	no	no	no	no	no
Year of current cancer diagnosis	2016	2016	2016	2015	2015	2015
Current cancer state (previous cancer diagnoses: type, year)	second cancer diagnosis (breast cancer right, 1970s)	first occurrence, in remission	first occurrence	first occurrence, in remission	first occurrence, in remission	first occurrence, in remission
Further relevant somatic illnesses	unknown	allergy	unknown	unknown	structural changes cervical spine; arterial hypertension; stomach burning	no
**Oncological treatment**						
Operation	x			x	x	x
Radiotherapy	x			x		
Chemotherapy		x	x		x	x
Hormonal treatment	x				x	x
Immunotherapy			x			
**Physical wellbeing**						
Body Appreciation Scale ^d)^ [sum score]	56	53	46	61	58	51
**Comments**						
	▪ “Previously, the body was like an envelope; currently (since retirement), it feels like it is about to become filled like a container” ▪ Substantial uncertainty about being a woman	▪ Ambiguous feelings regarding having a body, mostly driven by thoughts ▪ Conflicting attitudes regarding body image and age that are attributed to him by others based on his physical appearance	▪ High level of existential uncertainity in life and regarding the body	▪ Lack of confidence in the body	▪ Putting too many physical demands on herself	▪ Unfamiliar with limited bodily functioning ▪ Irritating feelings related to being a woman, which started with the bodily changes that occurred due to cancer

*Abbreviations: BAS, Body Appreciation Scale; ECOG, Eastern Cooperative Oncology Group* *Footnotes:* *^a)^ Distress thermometer ^61^: visual analog scale, ranging from not stressed = 0 to extremely stressed = 10* *^b)^ Selected items from the basis documentation for psycho-oncology ^60^, scale from 0 to 4: 0 = not at all; 1 = slightly; 2 = moderate; 3 = much; 4 = very much* *^c)^ Performance score of the ECOG ^63^; 0 = fully active, able to perform all pre-disease tasks without restriction; 1 = restricted in physically strenuous activity but ambulatory and able to perform light or sedentary tasks, e.g., light house work, office work; 2 = ambulatory and capable of all self-care but unable to carry out any work activities; up and about more than 50% of waking hours; 3 = capable of only limited self-care; confined to bed or a chair more than 50% of waking hours; 4 = completely disabled; cannot perform any self-care; completely confined to bed or a chair; 5 = dead* *^d)^ Validated German version of the 13-item BAS ^62^, with higher scores reflecting greater body appreciation*

The key statements of the patients are reported in Table 2 (for privacy protection, the statements are collapsed across patients). Notably, specific descriptions of progress showed substantial heterogeneity across subjects. Most patients indicated that sensations, perceptions, and other mental activities related to their own body intensified throughout the group BPT sessions.

**Table 2.** Statements during the initial individual session and six group sessions made by the patients regarding symptoms, main concerns, and other topics ^a)^.

**Initial individual** **session**	**Physical distress**: ▪ Persistent fatigue that impacts daily living and activities ▪ Polyneuropathy ▪ Persistent pain ▪ Loss of body parts ▪ Sexual dysfunction **Bodily perception of being stressed:** ▪ High level of uncertainty in life and unusual bodily feelings (e.g., bodily non-wellbeing, feeling of loss of body integrity, loss of function) ▪ Irritation related to gender identity; loss of the perception of being attractive	**Psychological distress & related resources** ▪ Sleep disturbance ▪ Mood swings (including initial shock) and worries ▪ Cognitive impairment: problems concentrating ▪ Fear of relapse ▪ Feeling irritable (e.g., inner pain of loss, search for meaning) ▪ Feelings of depression/sadness ▪ Feelings of guilt (e.g., pertinent question: Is there a relationship between stress and disease?) ▪ Shame/loss of self-esteem and feelings of helplessness/despair ▪ Good social support by family and friends ▪ Disease allows a break in professional activities, enabling one to reflect on one’s life	**Psychosocial problems** ▪ Divorce from partner and related separation from children ▪ Lack of intimate relationships ▪ Work-related problems (e.g., returning to work, daily conflicts, professional reorientation) ▪ Social withdrawal and isolation	**Further stressful** **factors** ▪ Problems with physicians (e.g., feelings of not being understood) ▪ Long medical history preceding cancer diagnosis
**1) General** **introduction,** **fostering of group** **cohesion and** **focus on bodily** **perception**	▪ Feelings of being relaxed: e.g., “ *I feel more relaxed after the exercise*”; “ *The body scan seems to be a good exercise at home*” ▪ Feelings of being welcomed: e.g., “ *I feel very welcome in the group”; “I felt very happy”* ▪ Body awareness: e.g., *“I realize that I have better bodily awareness than previously expected*”; “ *I was able to be aware of my body without* *focusing on the pain*” ▪ Feelings of agility: e.g., *“I was more awake than at the beginning of the session”*			
**2) Focus on bodily** **resources and** **grounding**	▪ Bodily perception of being stressed: e.g., “ *Persistent fatigue causing impairment in daily life and irritable mood*” ▪ Body awareness: e.g., *“I start feeling my feet*” ▪ Reduced psychological distress: e.g., “ *The exercises help me to fall asleep faster”;* “ *With the body awareness exercise, the ruminations stop* *earlier”* ▪ Enhanced psychological distress: e.g., *“Comparisons and exchanges with other group members foster my reflections on my own physical abilities;* *they bring about feelings of grief because I always lag behind during the exercises due to my persistent (non-cancer-related) physical impairment”* ▪ Psychosocial issues: e.g., “ *Restart and reorientation at work causes distress*”			
**3) Focus on** **closeness** **and distance** **regulation**	▪ Psychological resources: Gaining individual new knowledge about one’s own space: e.g., *“I feel the space that I need; I will use this to allow for* *more time and to be more aware of myself, to be conscious of my needs and to seek opportunities to recharge my batteries”* ▪ Gaining new individual knowledge about one’s boundaries: e.g., “ *Boundaries have a function; I feel safe and secure enough to see them more* *clearly now*”; “ *My boundaries help me to feel better and to stay in closer contact with myself*” ▪ Psychological distress: e.g., *“Expressing my needs was not easy; in particular, it was intimidating to see how others were easily able to be aware of* *their own space*”; “ *I realize that I do not want to have boundaries around me; the boundaries narrowed my space*”			
**4) Focus on social** **interactions and** **bodily impulses**	▪ Reduced psychological distress: e.g., *“I have done something new: saying no to others, respecting my need for my own space”* ▪ Enhanced psychological bodily distress, refuse the exercise, e.g., “ *This kind of movement does not correspond to my feelings and will*” ▪ Open questions: e.g., “ *How my space can be transported into daily life – e.g., when the phone is ringing at inconvenient times”* ▪ Psychosocial problem: e.g., “ *Moving out of my family home is challenging for me; therefore, having my own space and being separated and lonely* *at the same is difficult. How can I deal with this discrepancy*?” ▪ Awareness & presence: e.g., “ *I was able to maintain my bodily presence in my daily life*”; “ *I felt very satisfied with successfully staying present in* *my body during a challenging situation”*			
**5) Focus on** **embodied** **emotions**	▪ Awareness: e.g., “ *I feel a change in anticipated burden from the fatigue; I try to stay active or perform one of the exercises*” ▪ Psychological bodily distress and recourse: Addressing emotions: e.g., “ *It took me a long time to speak about personal feelings such as shame*”; “ *Anger was a strong feeling*” ▪ Psychological bodily distress: e.g., “ *When I feel excessive demands, I start feeling dizzy*” ▪ Observer position: e.g., “ *I realize that I have to build up my boundaries during this exercise*”			
**6) Summary and** **transfer session**	▪ Psychological distress and recourse: e.g., “ *It took courage being part of this group and therefore being highly visible”; “Seeing others with similar* *feelings helped me to understand my own processes of how to cope with this fate; I have learned to observe myself from a new perspective*” ▪ Self-contact and self-esteem: e.g., “ *The most important person in my life is me”*			

The statements reported here illustrate the most relevant topics and statements made by the participants throughout the therapeutic process. The collection is based on written notes from the group psychotherapist recorded during and directly after the sessions. *Footnote: ^a)^ To protect the participants’ privacy, the statements are reported without assigning them to individual patients.*

### Summary of main therapeutic processes and statements emerging throughout the group BPT sessions and at the end of the therapy

• At the beginning of the group BPT, a majority (5/6) of the participants referred to feelings of being left alone and partially helpless with the disease. Disturbances of bodily wellbeing and feelings of insecurity were commonly reported (4/6).
• At the end of the six BPT sessions, most participants reported improvements in wellbeing (5/6). They mentioned being more aware of physical and emotional boundaries and, therefore, having better knowledge of their coping strategies in conjunction with stress reduction in daily life (5/6). One participant referred to the observation of having gained a new sense of wholeness between body and soul.
• In response to the question “what has been supportive and what has felt effective”, most (5/6) patients stated that they enjoyed the exchange between cancer patients and learning about similar or even completely different experiences related to cancer. Others (3/6) mentioned having time and room to explore bodily wellbeing. One subject mentioned the solidarity and empathy within the group.
• All participants reported feeling comfortable (4/6) or very comfortable (2/6) during the group sessions.
• All participants reported that they felt they were being taken seriously and were supported, and they reported having enjoyed participating in the sessions.
• The majority of the participants (4/6) reported that they were neither under- nor over-challenged by the sessions. One participant reported that the movement sessions were challenging due to unfamiliarity, and one person with serious hearing problems was challenged in understanding and following exercises without direct visual contact.
• In response to the question “what was difficult for you”, half (3/6) of the participants replied that their perception was that not all participants had the same willingness or readiness to share their experiences in the group context. One participant stated not having felt a need to perform movement-related exercises, and, therefore, the related sessions were perceived as being too numerous.
• All participants reported that they would recommend this group BPT intervention to other patients.
• With regard to their satisfaction with the number of sessions provided, half of the participants (3/6) were satisfied with six sessions, and half of the participants (3/6) wished to have a minimum of two more sessions (one subject would have preferred 12 sessions).
• No adverse or unanticipated events were reported. All participants were able to ask questions or formulate their concerns. One subject stated that the room was too cold.

## Discussion

Despite the subjects having undergone different types of medical treatment for different cancer types and locations, all reported having appreciated the intervention and having progressed in how they perceived their body.

Most of the participants stated that they felt ready to address the bodily dimension of the experience of being affected by cancer and its treatments. The participants also appreciated that the group BPT offered space for them to achieve a new level of experiencing themselves from new perspectives. There were indications that the (bodily) presence of participants was enhanced across the group BPT sessions.

Nevertheless, group attendance and confrontation with one’s body (feelings and expression) were sometimes challenging and triggered temporary uncertainty in some subjects. Sharing these and other (bodily) experiences was helpful for integrating the bodily experiences.

The structure of the group BPT appeared to be appropriate to achieve the intended goals. Addressing body experiences and vulnerability following the experience of cancer was highly appreciated in this population. The participants felt positively about gaining new perspectives with regard to their bodily sensations and becoming more aware of relationships between thoughts, emotions and bodily actions, these findings are in line with previous reports (e.g. 64) Despite the limited duration of the intervention, patients appeared to transfer this new knowledge into actions in their daily life.

### Strengths and limitations

This case series has several strengths. To the best of our knowledge, this is the first systematic case report on group BPT for cancer patients. The study participants showed certain heterogeneity regarding age, gender, and cancer type, increasing the generalizability of the findings. Furthermore, we collected information not only on the overall subjective outcomes, but also on the perception of therapeutic processes. There are also several limitations that should be noted. We did not systematically assess how patients reacted to specific body psychotherapy interventions, which however would have been interesting, for example with regard to current efforts to develop individualized/personalized modular interventions. Further, we only collected information up to the end of the group BPT intervention. Future studies should also include follow-up assessments. Furthermore, application of established assessment instruments will provide important additional and complementary information on therapeutic efficacy of the intervention. Last, additional information, such as data collected via video- or audio-taping, will provide additional methods for exploring therapeutic processes related to the group BPT in more detail.

With regard to future studies, the observed heterogeneity in individual descriptions of perceived treatment effects point to the need to select rather comprehensive indicators of changes in disturbances of bodily wellbeing as a primary patient-reported outcome in future clinical trials. Notably, we here provide information on short-term body psychotherapy. Comparing the effects and their sustainability of short- and long-term body psychotherapy should be addressed in future studies. Moreover, patients reporting on group BPT triggering changes with regard to the meaning in life and other existential topics encourage more detailed exploration of this domain. Furthermore, linking and reconciling integrative BPT approaches, such as ‘integrative body therapy’ that assumes a ‘selfreflexive socioculturally embedded subject’, with recent insights from the fields of neuroscience and psychobiology, may be essential, though challenging, to fully exploit the current understanding and newly gained knowledge of cancer-related disturbances in bodily wellbeing and related interventions ^16, 65– 68^.

## Conclusion

The findings from this case series encourage and inform future studies aimed at identifying whether group BPT is efficacious in post-treatment cancer patients and related mechanisms of action. The observed heterogeneity in individual descriptions of detailed treatment effects point to the need to select rather comprehensive indicators of changes in disturbances of bodily wellbeing as a primary outcome in future clinical trials. While increases in mental activities related to one’s own body are commonly interpreted as an important mechanism of therapeutic action, follow-up assessments are needed to evaluate the intended, as well as unintended, consequences of these increases.

## Consent

Each patient provided written informed consent to report his/her case, including clinical and diagnostic information within this case series.

## Ethics statement

Ethical clearance was acquired from the *Ethikkommission Nordwest- und Zentralschweiz* in Basel, Switzerland (EKNZ) (EKNZ BASEC Req-2017-00513).

## Data availability

All data underlying the results are available as part of the article and no additional source data are required.

## Supplementary material

Supplementary file 1: Outline of the content of the group BPT sessions
