# Supplementary material for: A case series report of cancer patients undergoing group body psychotherapy
Source: F1000Res. 2018 Sep 5;6:1646. Originally published 2017 Sep 5. [Version 2] doi: 10.12688/f1000research.12262.2 (PMC5635441; doi:10.12688/f1000research.12262.2)
Supplement: Supplementary file 1 [file f1000research-6-17581-s0000.tgz › 024f724e-212f-4124-8531-4fc9e97d1875.docx]

Supplemental Material. Table: Outline of the content of the group BPT sessions.

| Sessions: Number and focus/topic | Session content |  |
| --- | --- | --- |
| 1. General introduction, fostering of group cohesion, and bodily perception | - Round of introductions with motivation, expectations and concerns regarding BPT - Reflections on bodily perceptions and current body image - Exercises: Breath perception; Body Scan (extended version); Being in contact with my body (hands on/off) | |
| 1. Bodily resources and grounding | - Introductory exercise: Breath perception - Balance between distress and resources, bodily stress reaction - Exercises: Body Scan, upright position; Grounding and anchoring; Movement exercise, including mirroring | |
| 1. Closeness and distance regulation | - Introductory exercise: Body scan (short version) and anchoring - Relevance of having own space and the choice between own and shared space - Exercises: Breathing techniques; Breathing space and fostering impulse to create own space; Exploring my own space | |
| 1. Social interactions and bodily impulses | - Introductory exercise: Body scan (short version); Breathing techniques; Creating own space - Awareness of bodily perception and the nature of impulses - Exercises: Imagination of ‘safe place’; Perception and awareness of body dimension; Awareness towards different body parts, own energy level, and own boundaries | |
| 1. Embodied emotions | - Introductory exercise: Body scan (short version) and anchoring - Connection between emotions, thoughts, actions, bodily sensations - Exercises: Embodiment: body sculptures of emotions; Following own bodily impulses and creating my own bodily space | |
| 1. Summary and transfer | - Introductory exercise: Body scan (short version) - Free choice of exercises to be repeated - Exercise to collate all experiences made during the past sessions - Summary and open questions | |
