# Supplementary material for: A case series report of cancer patients undergoing group body psychotherapy
Source: F1000Res. 2018 Sep 5;6:1646. Originally published 2017 Sep 5. [Version 2] doi: 10.12688/f1000research.12262.2 (PMC5635441; doi:10.12688/f1000research.12262.2)
Supplement: Peer review history

# Peer review: Referee response for version 1

**DOI:** 10.5256/f1000research.13275.r25724

**Published:** 2017-10-10

**Authors:** Manfred Thielen

**Affiliations:** 1 Institute for Body Psychotherapy, Berlin, Germany

The article is a clearly structured report on the results of the study and as such is both coherent and comprehensible. The scientific framework and the socio-demographic aspects of the study are exactly specified.

 The findings of the body psychotherapeutic group therapy with cancer patients are exceedingly interesting, particularly as the group therapy was a short-term therapy of only 6 sessions. It is clear from the combined, differentiated statements of the group participants that all of them have profited from the therapy, especially from the body psychotherapeutic approach. They have all attained new insights into their perceptions of body sensations and the relationship between thoughts, feelings and body activities. 5 of the 6 patients stated that their awareness of their physical and emotional borders had improved and that they are therefore able to reduce stress in their daily lives.

 The study shows impressively and probably for the first time, that this body psychotherapeutic group therapy with cancer patients has had very positive results.

 Of course these first positive results will have to be confirmed and elaborated by further studies. Since Wilhelm Reich (1948, 2001) there have been several approaches in body psychotherapy to the study of the origins and therapy of cancer, but the aspect of group therapy has not until now been examined. This study does therefore have an important pioneering aspect.

 In the light of the its merits my critical comments of the study are of minor significance. But I would have wished that the exact interventions and exercises, and their origins in which body psychotherapy approach, had been specified. It would also have been interesting to learn how the patients reacted to specific body psychotherapy interventions.

 Also I have some questions to the short-term-setting of the study. From my own experience over many years as leader of body psychotherapeutic groups, I see groups as having a more sustained long term effect if they take place over 2-3 years than if they are short-term.

I have read this submission. I believe that I have an appropriate level of expertise to confirm that it is of an acceptable scientific standard.

## References

[1] ReichW.: Entdeckung Des Orgons. *Orgone Inst. Press.*.1948, 2001;1:

[2] ReichW.: Discovery of the Orgone, Volume II: The Cancer Biopathy. 1973;

# Response

**Published:** 2018-08-23

**Authors:** Gunther Meinlschmidt

**Affiliations:** Department of Clinical Psychology and Epidemiology, University of Basel, Switzerland

**Dear Dr. Thielen,**

**We thank you very much for providing us with insightful and constructive reviewer comments on our article. We would like to take the opportunity to reply to your comments and provide a revised version. We considered all points raised and include our point-by-point responses below:**

**Comment 1:** “In the light of the its merits my critical comments of the study are of minor significance….”

*We thank the reviewer for the overall positive feedback.*

**Comment 2:** “I would have wished that the exact interventions and exercises … had been specified.”

*We agree with the reviewer that more information on the interventions and exercises would be an asset. We therefore plan to publish an in depth description of the intervention as separate publication in the upcoming year. We thereby intend to take advantage of F1000 articles as* *‘living’ („even after peer review is complete: Authors can ‘update’ their articles at any time (and at no extra charge) if there have been small developments relevant to the findings“) and will insert into the article a link to this separate publication as soon as possible.*

**Comment 3:** “I would have wished that (…) their origins in which body psychotherapy approach, had been specified.”

*The concept of this group body psychotherapy approach is rooted in the integrative body psychotherapy (IBP) movement, conceptualized by Jack Lee Rosenberg. The IBP approach is described in more detail in reference 42. In the revised version of the manuscript, we have added an additional reference (Rosenberg, J.L., Rand, M. L. and Asay, D. (1985); Body, Self, and Soul. Sustaining Integration. Humanics Limited, Humanics New Age; Atlanta, Georgia) in which the origins are further outlined.*

**Comment 4:** “It would also have been interesting to learn how the patients reacted to specific body psychotherapy interventions”

*We completely agree with the reviewer that information on how the patients reacted to specific body psychotherapy interventions would be highly interesting. Unfortunately, we didn’t collect respective data this time, but intend to do so in future studies. We added a respective sentence in the discussion section of our manuscript:*

*“We did not systematically assess how patients reacted to specific body psychotherapy interventions, which however would have been highly interesting, for example with regard to inform current efforts to develop more modular, individualized/personalized modular interventions.”*

**Comment 5:** “Also I have some questions to the short-term-setting of the study. From my own experience over many years as leader of body psychotherapeutic groups, I see groups as having a more sustained long term effect if they take place over 2-3 years than if they are short-term.”

*We fully agree that whether short- or long-term body psychotherapy interventions for cancer patients show better and more sustained effects is a very interesting question, which however goes beyond the scope of this project. We added a respective sentence in the discussion section, highlighting that this is a relevant issue for further studies:*

*“Notably, we here provide information on short-term body psychotherapy. Comparing the effects and their sustainability of short- and long-term body psychotherapy should be addressed in future studies.”*

# Peer review: Referee response for version 1

**DOI:** 10.5256/f1000research.13275.r25718

**Published:** 2017-09-15

**Authors:** Ulrich Sollmann

**Affiliations:** 1 German - Chinese Academy for Psychotherapy, Heidelberg, Germany

Goal, structure and results of the report are described very clearly. The report is comprehensible, understandable and verifiable. The results include enough data about the socio-demographic aspects in medical history etc. The results mirror also, and this is very important, individual and personal statements which are related to the individual sessions in the group as well as to the individual patients and their concerns. The combination of both kinds of results seems to be a helpful illustration so that somebody who didn’t join the group can imagine better what the patients experienced in the group; personally and bodily. This also gives a deep inside into the therapist`s perspective, the therapist’s awareness and perception.

 The structure (six sessions in the group) is short and long enough to establish a process of body self-experience. The group-setting invites to get a really competent overview of what had happened and why it happened.

 I guess that the distinction between positive effects on the group-level in comparison with the individually perceived treatment could be very interesting for future research.

 It’s remarkable that the authors refer elaborated on various literature of the field of body-psychotherapy. This could be an important step to better integrate body-psychotherapy in the field of psychosomatic medicine. What is urgently needed.

 There are some remarks I want to add and which could be considered in future research:

- It could be helpful to define the specific concept of body-psychotherapy in the group and thus included relevance of psycho-educative aspects.
- It would be very helpful to get a better understanding of a specific concept of body-psychotherapy which was used in the group. The field of body-psychotherapy offers a very big variety of approaches in body-psychotherapy. It could be helpful to better understand the background. This understanding could be necessary to better understand and interpret the results of such a study.
- According to the literature on working with body-psychotherapy in the group with cancer-patients there is more literature being available of course for many years. It could be interesting to discuss the results of such a study on the background of scientific research in the period of the last 25 years.

I have read this submission. I believe that I have an appropriate level of expertise to confirm that it is of an acceptable scientific standard.

# Response

**Published:** 2018-08-23

**Authors:** Gunther Meinlschmidt

**Affiliations:** Department of Clinical Psychology and Epidemiology, University of Basel, Switzerland

**Dear Dr. Sollmann,**

**We thank you very much for providing us with insightful and constructive reviewer comments on our article. We would like to take the opportunity to reply to your comments and provide a revised version. We considered all points raised and include our point-by-point responses below:**

*We thank the reviewer for the overall positive feedback*

**Comment 1:** It could be helpful to define the specific concept of body-psychotherapy in the group and thus included relevance of psycho-educative aspects.

*We agree with the reviewer that more information on the interventions and exercises would be an asset. We therefore plan to publish an in depth description of the intervention as separate publication in the upcoming year. We thereby intend to take advantage of F1000 articles as* *‘living’ („even after peer review is complete: Authors can ‘update’ their articles at any time (and at no extra charge) if there have been small developments relevant to the findings“) and will insert into the article a link to this separate publication as soon as possible.*

**Comment 2:** It would be very helpful to get a better understanding of a specific concept of body-psychotherapy which was used in the group. The field of body-psychotherapy offers a very big variety of approaches in body-psychotherapy. It could be helpful to better understand the background. This understanding could be necessary to better understand and interpret the results of such a study.

*The concept of this group body psychotherapy approach is rooted in the integrative body psychotherapy (IBP) movement, conceptualized by Jack Lee Rosenberg. The IBP approach is described in more detail in reference 42. In the revised version of the manuscript, we have added an additional reference (Rosenberg, J.L., Rand, M. L. and Asay, D. (1985); Body, Self, and Soul. Sustaining Integration. Humanics Limited, Humanics New Age; Atlanta, Georgia) in which the origins are further outlined.*

**Comment 3:** According to the literature on working with body-psychotherapy in the group with cancer-patients there is more literature being available of course for many years. It could be interesting to discuss the results of such a study on the background of scientific research in the period of the last 25 years.

 We thank the reviewer for pointing this out and expanded the discussion section:

*“ these findings are in line with previous reports (e.g. 64) “*
